# A hybrid fuzzy logic–Random Forest model to predict psychiatric treatment order outcomes: an interpretable tool for legal decision support

**DOI:** 10.3389/frai.2025.1606250

**Published:** 2025-06-17

**Authors:** Alexandre Hudon

**Affiliations:** ^1^Centre de recherche de l’Institut universitaire en santé mentale de Montréal, Montreal, QC, Canada; ^2^Department of Psychiatry, Institut universitaire en santé mentale de Montréal, Montreal, QC, Canada; ^3^Department of Psychiatry, Institut national de psychiatrie légale Philippe-Pinel, Montreal, QC, Canada; ^4^Department of Psychiatry and Addictology, Université de Montréal Faculty of Medicine, Montreal, QC, Canada

**Keywords:** fuzzy logic, Random Forest, psychiatric treatment orders, legal decision support, forensic psychiatry, machine learning, interpretability, law

## Abstract

**Background:**

Decisions surrounding involuntary psychiatric treatment orders often involve complex clinical, legal, and ethical considerations, especially when patients lack decisional capacity and refuse treatment. In Quebec, these orders are issued by the Superior Court based on a combination of medical, legal, and behavioral evidence. However, no transparent, evidence-informed predictive tools currently exist to estimate the likelihood of full treatment order acceptance. This study aims to develop and evaluate a hybrid fuzzy logic–machine learning model to predict such outcomes and identify important influencing factors.

**Methods:**

A retrospective dataset of 176 Superior Court judgments rendered in Quebec in 2024 was curated from SOQUIJ, encompassing demographic, clinical, and legal variables. A Mamdani-type fuzzy inference system was constructed to simulate expert decision logic and output a continuous likelihood score. This score, along with structured features, was used to train a Random Forest classifier. Model performance was evaluated using accuracy, precision, recall and F1 score. A 10-fold stratified cross-validation was employed for internal validation. Feature importance was also computed to assess the influence of each variable on the prediction outcome.

**Results:**

The hybrid model achieved an accuracy of 98.1%, precision of 93.3%, recall of 100%, and a F1 score of 96.6. The most influential predictors were the duration of time granted by the court, duration requested by the clinical team, and age of the defendant. Fuzzy logic features such as severity, compliance, and a composite Burden_Score also significantly contributed to prediction accuracy. Only one misclassified case was observed in the test set, and the system provided interpretable decision logic consistent with expert reasoning.

**Conclusion:**

This exploratory study offers a novel approach for decision support in forensic psychiatric contexts. Future work should aim to validate the model across other jurisdictions, incorporate more advanced natural language processing for semantic feature extraction, and explore dynamic rule optimization techniques. These enhancements would further improve generalizability, fairness, and practical utility in real-world clinical and legal settings.

## 1 Introduction

In psychiatric care, particularly in acute and forensic contexts, there are situations in which patients are incapable of consenting to treatment and simultaneously refuse all interventions, including psychotropic medication. These scenarios present complex clinical and ethical challenges, as the refusal of care may result in self-harm, deterioration of mental status, or threats to others. In such cases, legal mechanisms like treatment orders become essential tools to ensure continuity of care while safeguarding civil liberties (Saya et al., 2019; Szmukler and Holloway, 1998). These orders permit the administration of treatment without full consent when patients are found to lack decisional capacity due to psychiatric illness such as schizophrenia, bipolar disorder with psychotic features, or severe depressive episodes with psychosis (Pons et al., 2020; Owen et al., 2008). The use of treatment orders reflects a delicate balance between patient autonomy and the necessity for care in the face of incapacity (Bergamin et al., 2022).

In Quebec, treatment orders are issued under the jurisdiction of the Superior Court, following legal frameworks established in the Civil Code of Quebec and applicable jurisprudence. The legal process typically begins when a treating psychiatrist summons the court, supported by documentation that the patient lacks capacity and that treatment is urgently required (O'Reilly et al., 2019). The patient is afforded the right to contest the application and is often represented by legal aid counsel. The judge considers expert testimony, diagnostic assessments, and social context before determining whether to authorize the treatment order, in part or in full (Frank et al., 2020). The decision itself is not a simple clinical translation. It reflects a legal interpretation of psychiatric risk, patient history, compliance patterns, and the proportionality of the request. As such, decisions can vary significantly across judges and institutions despite similar clinical contexts (Rugkåsa, 2016; Weich et al., 2020).

Given the subjective, cross-disciplinary, and time-sensitive nature of these legal decisions, identifying the important variables that influence a judge’s decision to fully accept a treatment order is both clinically and legally relevant. However, the semi-structured nature of psychiatric records and the complex language of legal documentation pose barriers to standard predictive modeling (London, 2019). Furthermore, traditional machine learning models often lack interpretability, making them less suited for ethically sensitive applications in mental health law (Goktas and Grzybowski, 2025; Ennab and Mcheick, 2024). Fuzzy logic systems, by contrast, allow for expert rule-based reasoning that accommodates uncertainty and vagueness which are common characteristics in psychiatric data (Torres and Nieto, 2006; Shoaip et al., 2024; Modai et al., 2004). When combined with Random Forests, an ensemble-based machine learning method known for handling non-linear relationships and high-dimensional data, fuzzy logic can enhance model performance without sacrificing interpretability (Hao et al., 2022; Shoaip et al., 2024).

The objective of this proof-of-concept, exploratory study is twofold. First, it aims to build a predictive model that accurately estimates the probability that a Superior Court in Quebec will accept a psychiatric treatment order as requested by the treating team. Second, it seeks to identify the most influential features contributing to that outcome, including demographic variables, legal context, clinical diagnosis, and behavioral compliance. It is hypothesized that features such as diagnosis severity, time requested by the treating team, non-compliance behaviors, and substance use history will emerge as significant predictors. By integrating expert-driven rule logic with empirical learning, the model aims to support clinicians, legal professionals, and policymakers in making more transparent, equitable, and evidence-informed decisions.

## 2 Materials and methods

### 2.1 Description of the dataset

This study draws on a manually curated dataset titled *TreatmentOrderQuebec2024*, comprising real-world data on mental health treatment order requests rendered in Quebec during the year 2024 from January 1st to December 31st. More specifically, it comprises the 176 judgments from the Superior Court that were identified in the publicly sponsored legal information service in Quebec, Société québécoise d’information juridique (SOQUIJ) (Société québécoise d'information juridique, n.d.). It is important to note that SOQUIJ anonymizes each case reported publicly in their database. Each entry represents a legal case evaluated by the Superior Court and includes both structured variables and semi-structured clinical text. The dataset contains several variables encompassing demographic characteristics (such as the age and sex of the defendant), legal context (including whether the defendant had legal representation or was assisted by legal aid), and clinical indicators (such as primary and secondary diagnoses, behavioral symptom descriptions, and substance use history). It also captures procedural information, notably the duration of treatment requested by the clinical team, the duration granted by the court, and the outcome: specifically, whether the order was accepted in full or only partially granted. The characteristic reported in the dataset are found in Table 1. The main outcome variable used for model training and evaluation is a binary indicator reflecting the court’s decision to either entirely or partially accept the treatment order. To ensure validation, the dataset was split into training and testing subsets using a 70/30 ratio, with 70% of the data reserved for model development and 30% held out for independent performance assessment (Collins et al., 2024). The dataset is available as Supplementary Material 1.

**Table 1:** Summary of the variables and their definitions.

Variables	Definitions
Was the defendant represented?	Was the defendant represented by a lawyer?
If represented was it by legal aid?	Indicates whether the legal representation was through public legal aid.
Age of the defendant	Age in years of the person subject to the treatment order request.
Sex of the defendant	Biological sex of the defendant (Male/Female).
Main diagnosis of the defendant	Primary psychiatric diagnosis documented by the clinical team.
Other diagnoses listed	List of comorbidities listed in the judgment.
Symptoms and signs	List of symptoms and signs listed in the judgment.
Was substance use reported?	Indicates whether substance use was mentioned in the case record.
If so, what are the substances listed	List of the substances found in the judgment.
Was it a treatment order request or was it treatment and housing?	Statement that highlights if it was only requesting a treatment order or treatment order plus housing.
What treatment is requested	Treatment request found in the judgment.
What are the accessory requests (e.g., blood samples, urinary tests, ECT, etc)	List of accessory requests provided in the judgment.
What was the time (in years) requested by the treating team	Treatment duration requested by the clinical team.
Was the request accepted or denied?	The decision of the judge.
Number of time granted (in years)	Treatment duration granted by the Superior Court.
If accepted, was it partially or entirely?	Final court decision on the treatment order (Partial/Entirely).

### 2.2 Fuzzy logic modeling

A fuzzy inference system (FIS) was developed using a Mamdani-type architecture to encode expert reasoning and produce interpretable predictions regarding the outcome of psychiatric treatment order requests (Guillaume and Charnomordic, 2012). Implemented in Python 3.11, this system was designed to output a continuous score, known as the fuzzy score, between 0 and 1 that represents the likelihood that a treatment order would be entirely accepted by the court. The system was created to mirror the reasoning patterns of mental health and legal experts, translating qualitative assessments (e.g., severity, compliance, context) into a structured, explainable logic model. This approach allowed for both nuanced classification and transparency in decision-making.

The fuzzy model was implemented using a combination of widely recognized Python libraries. Specifically, pandas and numpy were used for data handling and transformation, while scikit-learn supported preprocessing, scaling, and integration into the broader machine learning pipeline (Harris et al., 2020; The pandas development team, 2020). Fuzzy logic inference was handled via the scikit-fuzzy (skfuzzy) library, which provided tools for defining fuzzy variables, membership functions, and rule application (Pedregosa et al., 2011).

To enable fuzzification of key features, numerical variables were normalized to a common [0, 1] scale using Min-Max scaling. Triangular membership functions were then defined for core fuzzy inputs. For example, the “Age of the Defendant” variable was fuzzified into three linguistic categories: Young ([0.0, 0.0, 0.3]), Middle-aged ([0.2, 0.5, 0.7]), and Older ([0.8, 1.0, 1.0]). Similarly, both requested and granted treatment durations were described as Short ([0.0, 0.0, 0.3]), Medium ([0.2, 0.5, 0.7]), or Long ([0.6, 1.0, 1.0]). The fuzzy output variable, representing the Likelihood of Entire Acceptance, was categorized into three fuzzy sets: Low ([0.0, 0.0, 0.4]), Medium ([0.3, 0.5, 0.7]), and High ([0.6, 1.0, 1.0]).

Semantic features were derived through natural language processing of textual case data. Two binary flags were introduced to capture clinically meaningful patterns. The *Severity_Flag* was set to 1 if the main diagnosis field contained terms like “schizophrenia,” “psychosis,” or “bipolar disorder,” reflecting high diagnostic severity. The *Compliance_Flag* was similarly set to 1 if the behavioral description included language such as “non-compliance,” “refused,” or “discontinued,” indicating potential treatment resistance. These semantic flags played an important role in the fuzzy rule base, in order to offer binary anchors for more complex rule conditions.

Rather than assigning fixed classifications, the system employed a weighted rule-based approach to produce a fuzzy score. Each rule contributed a weighted score to the final output, allowing for partial activation across multiple rules. For instance, the rule “Severe + Non-compliant → High” carried a full weight of 1.0, whereas “Older + Severe + Compliant → Medium” was assigned a weight of 0.7. Other rules included “Low Severity + Compliance → Low” (weight: 1.0), “Young Male + Substance Use + Long Duration → High” (weight: 0.8), and “Female + High Burden Score → Medium-High” (weight: 0.6). This weighted logic allowed for dynamic blending of rule contributions based on individual case profiles, increasing both predictive power and interpretability. The ten rules implemented in this model are reported in Table 2.

**Table 2:** Rules of the fuzzy inference system.

Rule #	Condition	Predicted Likelihood	Weight
1	Severe diagnosis AND non-compliance	High	1
2	Severe diagnosis AND history of prior compliance	Medium	0,8
3	Low severity AND compliant	Low	1
4	Young male WITH substance use AND long duration requested	High	0,8
5	Older defendant WITH severe diagnosis AND compliance	Medium	0,7
6	Female defendant WITH high Burden_Score	Medium-High	0,6
7	Short treatment duration AND legal aid support	High	0,7
8	Severe diagnosis BUT short time requested AND no compliance issues	Medium	0,6
9	Mild/moderate diagnosis WITH non-compliance	Medium	0,5
10	Severe diagnosis WITH substance use AND long time requested	High	1

To further capture the multidimensional burden of each case, a *Burden_Score* was calculated as a weighted composite of the features. The weights were chosen based on clinical relevance and empirical signal strength: Severity (35%), Compliance (30%), Time Requested (20%), and Substance Use (15%). The resulting *Burden_Score* offered a normalized index of clinical and legal complexity, which could then be used directly in rules or passed downstream into machine learning classifiers.

The fuzzy inference system produced a continuous output referred to as the *Expanded_Score*, representing the degree of likelihood that the court would grant a treatment order in full. This score was derived by averaging the outputs of all activated rules, weighted by their respective importance. For interpretability, the *Expanded_Score* was categorized into three final risk levels using defuzzification thresholds: Low (< 0.4), Medium (0.4 to 0.7), and High (> 0.7). This final output served as a key input into the hybrid modeling stage, where it was integrated with structured features for supervised classification. The pseudocode for the implementation is found in Supplementary Material 2.

### 2.3 Random Forest classifier

The second stage involved training a Random Forest classifier using the combined dataset, which included both raw features and the fuzzy-derived *Expanded_Score*. The model was implemented using the *RandomForestClassifier* class from the *sklearn.ensemble* library, with the parameter *class_weight = ‘balanced’* to address potential class imbalance between cases that were entirely versus partially accepted (Pedregosa et al., 2011). The input features used for training included: age of the defendant, time requested by the treating team, time granted by the court, legal aid representation, sex, substance use status, semantic flags (severity and compliance), the *Burden_Score*, and the fuzzy *Expanded_Score*. The model was trained on 70% of the dataset and evaluated on the remaining 30% using both predicted labels and prediction probabilities.

To assess model interpretability and understand the relative influence of each variable, feature importance analysis was conducted using the Random Forest model’s *feature_importances_* attribute. This function computes the mean decrease in Gini impurity contributed by each feature, normalized across all predictors. A Gini impurity is a measure used in decision tree algorithms to quantify how often a randomly chosen element from a set would be incorrectly labeled if it were randomly assigned a label according to the distribution of labels in that set (Das et al., 2018).

### 2.4 Data analysis

Model performance was assessed through a suite of supervised classification evaluation techniques designed to measure predictive accuracy, class discrimination, and reliability. Class probabilities generated with *predict()* and *predict_proba()* from *sklearn.ensemble. RandomForestClassifier* (Pedregosa et al., 2011). The binary classification outcome (entirely vs. partially accepted treatment orders) was compared to the ground truth labels using four standard evaluation metrics: accuracy, precision, recall and the F1 score (Hicks et al., 2022). Accuracy measured the overall proportion of correct classifications, while precision focused on how many of the cases predicted as fully accepted were actually accepted in full, minimizing false positives. Recall assessed the model’s ability to correctly identify all truly accepted cases, ensuring no eligible cases were missed. The F1 score provided a balanced metric that accounts for both precision and recall, particularly useful in slightly imbalanced datasets.

To further analyze prediction patterns and error distribution, a confusion matrix was generated using confusion_matrix() and visualized with *seaborn* providing an intuitive view of true positives, false positives, and other classifications across the binary decision space (Waskom, 2021). Additionally, a correlation heatmap was computed using *pandas* and displayed with *seaborn* to explore linear relationships among relevant features, including the fuzzy output (*Expanded_Score*), *Burden_Score*, and the durations of treatment requested and granted. The choice of these variables was based on the fact that only numeric, monotonic predictors were eligible for a bivariate correlation table. All other predictors (e.g., sex, representation status, diagnosis categories) are nominal or ordinal and would have required point-biserial or rank-based statistics that complicate a single, readable table. With only 176 cases, adding many low-variance or categorical features to a correlation matrix inflates the familywise Type I error without providing interpretable effect sizes.

### 2.5 Cross-validation

A 10-fold stratified cross-validation procedure was employed during development (Jung and Hu, 2015). This method involved partitioning the training dataset into ten equally sized subsets, or folds, while maintaining the original distribution of the binary outcome (entirely versus partially accepted treatment orders). For each iteration, the model was trained on nine folds and validated on the remaining one, cycling through all combinations. Stratification ensured that class imbalance did not affect any fold disproportionately, thus preserving the fairness and reliability of the evaluation.

### 2.6 Ethical considerations

All ethical principles were upheld throughout this study. In terms of data privacy, all personal identifiers were removed in accordance with SOQUIJ’s database, and the dataset was processed in full compliance with applicable privacy standards. According to Tri-Council Policy Statement 2 on Ethical Conduct for Research Involving Humans, ethics approval was not required because all the material used to construct the dataset is publicly available (Conseil de recherches en sciences humaines du Canada, n.d.).

Finally, the model is intended strictly as an exploratory decision support tool rather than a substitute for professional judgment. Its use is to provide a proof-of-concept for enhancing risk stratification, guiding policy evaluation, and supporting procedural transparency in complex mental health adjudication contexts.

## 3 Results

### 3.1 Fuzzy logic output and categorization

**Figure 1:**
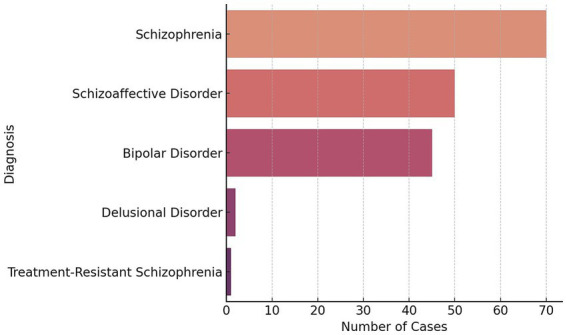
Top 5 diagnosis identified in the judgments.

Each sample in the dataset was first evaluated using the fuzzy logic layer, which implemented 10 expert-defined rules with weighted scoring. The top 5 diagnosis identified in the judgments are reported in Figure 1. These rules assessed key variables such as diagnosis severity, treatment compliance, the duration of treatment requested, patient age, sex, substance use, and legal aid involvement. The resulting fuzzy outputs (*Expanded_Score*) ranged between 0.3 and 0.85 and were defuzzified using the centroid method. Among the 53 cases in the test set, 56.6% were categorized as Medium and 43.4% as High, with no cases falling into the Low category.

### 3.2 Combined model performance

**Figure 2:**
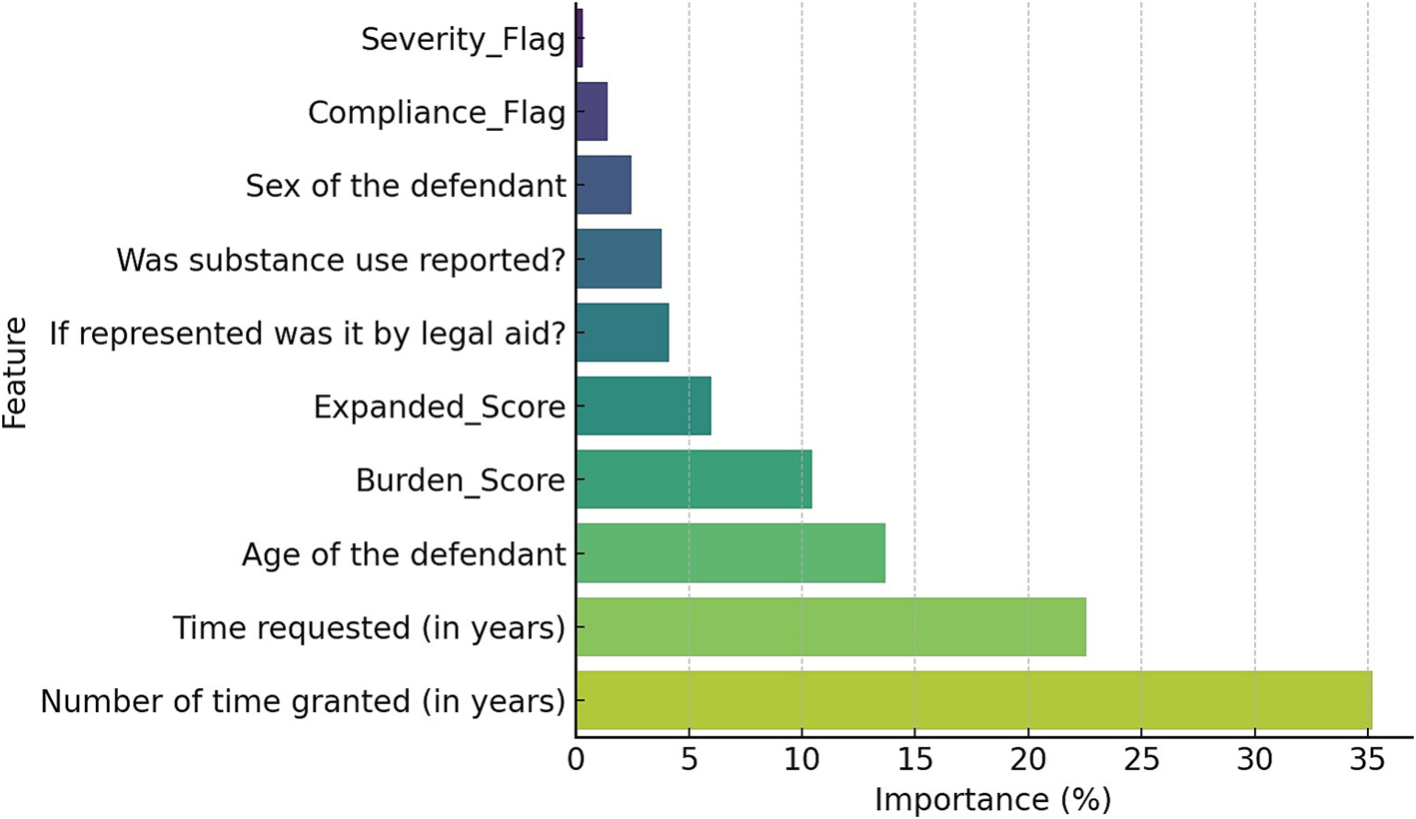
Importance of the combined model’s features.

The model achieved an accuracy of 98.1% (95% CI: 92.3–100%), precision of 93.3% (95% CI: 85.7–100%), recall of 100% (95% CI: 94.9–100%), and F1 score of 96.6% (95% CI: 91.2–100%). The confusion matrix revealed only one misclassified case among the 53 test instances which can be found in Figure 2. This indicates a strong alignment between predicted outcomes and actual court decisions.

**Figure 3:**
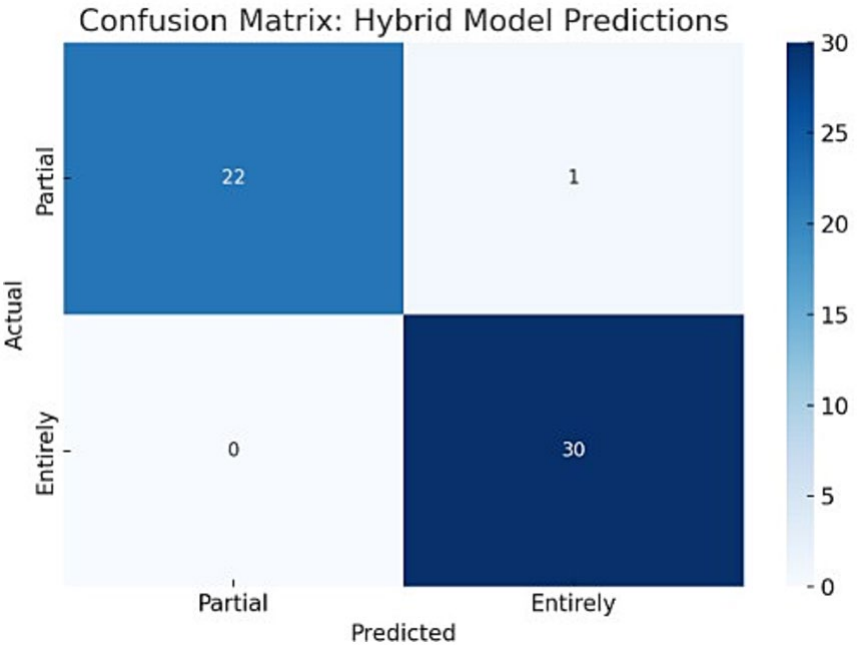
Confusion matrix of the hybrid model predictions.

The most influential predictor was the duration of time granted by the court (35.2%), followed by the duration requested by the treating team (22.6%) and the age of the defendant (13.7%). The Burden_Score contributed 10.4%, and the fuzzy system’s *Expanded_Score* added 6.0% to the overall model prediction. Semantic flags also played nontrivial roles. Features like legal aid, substance use, and sex were less predictive individually (<2%), but contributed contextually to the model’s overall structure. The importance of features is reported in Figure 3.

**Figure 4:**
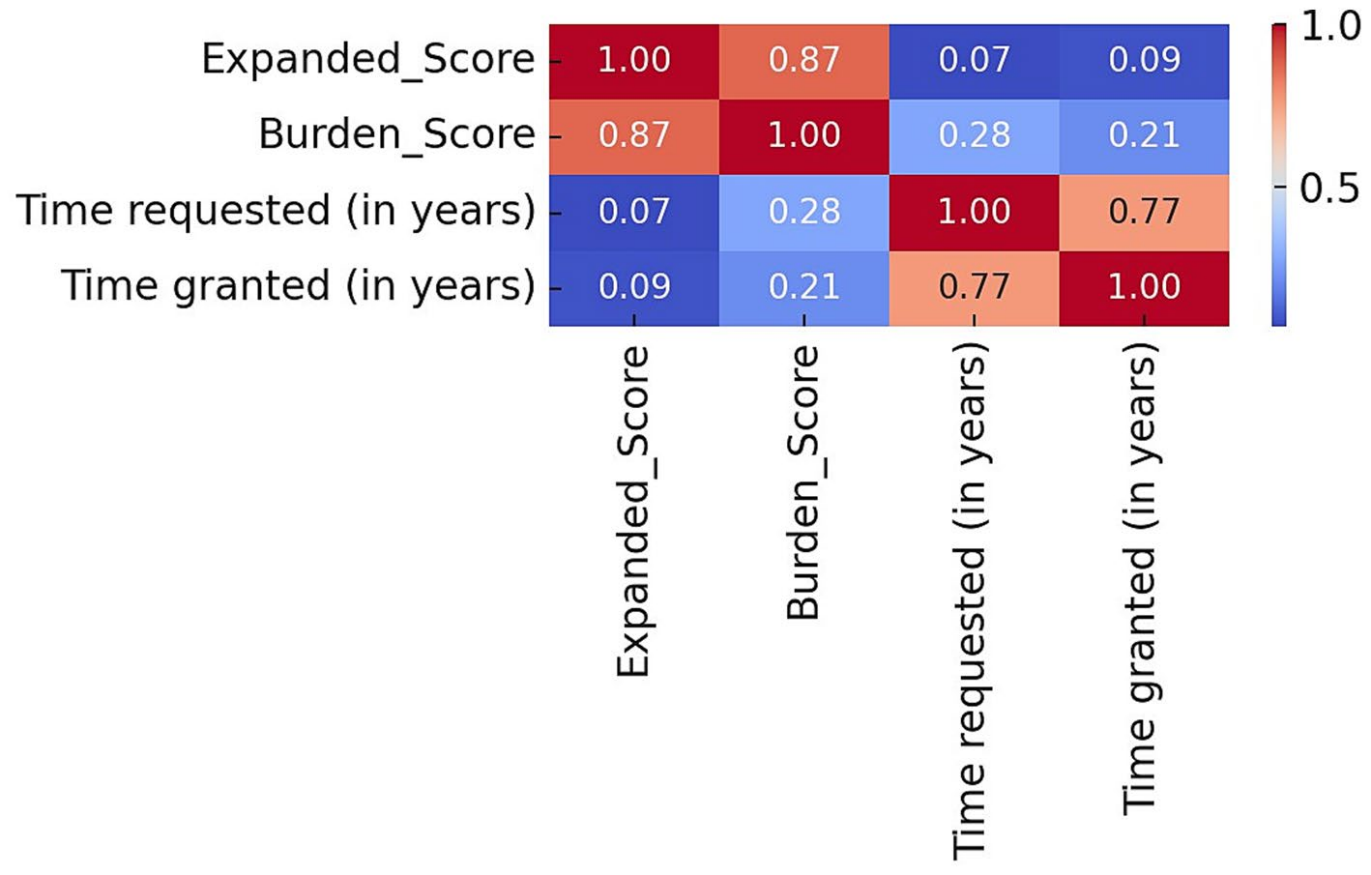
Correlation heatmap of fuzzy and model features.

The correlation heatmap revealed several meaningful relationships between the variables in the hybrid model. A strong correlation (r = 0.87) between the *Expanded_Score* and the *Burden_Score* suggests that the fuzzy system’s outputs are closely aligned with the composite clinical and legal complexity captured by the *Burden_Score*, which integrates inputs such as severity, compliance, substance use, and treatment duration. Similarly, a strong correlation (r = 0.77) between time requested and time granted indicates that judicial decisions tend to reflect the duration recommended by the clinical team. In contrast, the *Expanded_Score* showed very weak correlations with time requested (r = 0.07) and time granted (r = 0.09), suggesting that the fuzzy system operates independently of duration-based inputs and is more heavily influenced by rule-based clinical and behavioral features. The *Burden_Score* showed only modest correlations with time requested (r = 0.28) and time granted (r = 0.21), indicating that while duration contributes to the burden calculation, it does not dominate it which reinforces the multidimensional nature of the composite score. The heatmap is found in Figure 4.

### 3.3 Case-based reasoning and interpretability

To assess how the model performs on individual profiles, two illustrative cases were examined. In the first example, a patient diagnosed with schizophrenia who was non-compliant with treatment and recommended for a long treatment duration under legal aid received a fuzzy score of 0.85 and was correctly predicted to be entirely accepted. In the second case, a compliant patient with a mood disorder, a short treatment request, and legal aid representation received a fuzzy score of 0.45 and was correctly classified as partially accepted. These examples highlight how the model’s reasoning aligns with typical clinical and legal expectations.

## 4 Discussion

This study demonstrates the feasibility and potential utility of combining fuzzy logic with supervised machine learning to predict judicial decisions regarding psychiatric treatment orders. By integrating clinical, legal, and semantic information within a hybrid fuzzy–Random Forest framework, the model achieved strong predictive performance and offered interpretable outputs aligned with expert reasoning. These findings suggest that decision support tools grounded in hybrid modeling can offer value in ethically sensitive, cross-disciplinary contexts such as involuntary psychiatric treatment.

The high predictive performance observed suggests that the hybrid model is highly capable of distinguishing between cases that will be entirely versus partially accepted by the court. These results are important given the complexity and subjectivity of such decisions. Prior work in clinical decision support and mental health law has emphasized the limitations of purely statistical or black-box models in capturing the ambiguity of psychiatric documentation and judicial interpretation (Chekroud et al., 2021; McCradden et al., 2023). Traditional machine learning approaches often struggle to accommodate the vagueness and semantic variability inherent in mental health records (Holzinger et al., 2019). Fuzzy logic, by contrast, provides a formal framework to model uncertainty and expert knowledge, which is especially valuable in psychiatric decision-making (Torres and Nieto, 2006). In this study, the use of expert-defined fuzzy rules attempted to allow for transparent reasoning processes that mirror how judges and clinicians synthesize narrative and structured data.

The use of interpretability-focused AI is especially relevant in legal-medical settings, where decision accountability and traceability are essential. Several researchers have highlighted the importance of explainability in clinical AI to ensure trust and fairness, particularly in high-stakes domains (Abgrall et al., 2024; Asan et al., 2020). Sirocchi et al. emphasize that models used in medicine must not only be accurate but also interpretable by domain experts (Sirocchi et al., 2024). Similarly, Rama et al. advocate for hybrid models that balance statistical rigor with symbolic reasoning to ensure stakeholder acceptance (Raman et al., 2025). In the present study, the fuzzy inference layer played a role in ensuring model transparency. By encoding rules such as “Severe + Non-compliant → High likelihood of acceptance” the system provided logic paths that could be validated or critiqued by legal or clinical stakeholders, enhancing the model’s usability and trustworthiness.

In addition to predictive performance, the study contributes to a growing body of literature supporting hybrid symbolic-statistical models in healthcare and law. Holzinger et al. argue that hybrid approaches allow for “augmented intelligence” by integrating domain expertise into data-driven models (Holzinger et al., 2019). This is relevant in psychiatric contexts, where machine learning alone may miss important context-specific information that clinicians routinely interpret (Torres and Nieto, 2006). The present model’s ability to integrate fuzzy outputs with structured variables (e.g., age, treatment duration, legal aid status) and semantic indicators (e.g., compliance, severity) reflects this “augmented” approach. Importantly, the inclusion of the *Burden_Score* also aligns with literature advocating for holistic, multi-dimensional indicators in forensic psychiatry (Raharjanti et al., 2021).

Although legal frameworks may vary internationally, the core challenges (balancing patient autonomy, clinical risk, and procedural fairness) are widely shared (Mikellides et al., 2019). The decision-making process in Quebec’s Superior Court is informed not only by medical evaluations but also by judicial discretion shaped by case law and social values. Thus, modeling approaches must reflect both the evidence-based nature of psychiatry and the normative reasoning of law in the specific jurisdiction. The hybrid fuzzy–Random Forest model presented here offers a promising approach to capturing that intersection.

It is also important to note that the fuzzy inference system was explicitly designed to enhance interpretability via rule-based logic, the study did not include a formal expert evaluation of interpretability. Future work should engage legal and psychiatric professionals to assess whether the system’s explanations are clinically meaningful, cognitively accessible, and ethically appropriate for real-world decision support.

Finally, while the findings of this study are promising, several limitations must be acknowledged. The dataset was limited to 176 cases from Quebec’s Superior Court in 2024, which may constrain the generalizability of the model to other jurisdictions or legal systems with differing criteria for involuntary treatment. Furthermore, not all the treatment orders that took place in Quebec in 2024 were reported in the SOQUIJ’s database which may underrepresent certain areas. The dataset also lacked temporal or longitudinal data, such as prior treatment history or recidivism, which could enhance predictive accuracy. Also, the construction of semantic features such as severity and compliance flags relied on keyword matching from clinical narratives, which may not fully capture the nuance of psychiatric assessments and could introduce misclassification bias. Furthermore, although the fuzzy logic system was based on expert-informed rules, the weighting of those rules was manually tuned rather than optimized through formal learning algorithms, potentially limiting the model’s scalability and introduce overfitting. While the model achieved a recall rate of 100% in the held-out test set, this result should be interpreted in light of the relatively small sample size and the potential for overfitting. With only 53 cases in the test set, even a single misclassified instance would have led to a marked reduction in recall. As such, the recall score likely reflects model performance on this specific dataset and may not generalize to future or out-of-distribution cases. Future work should prioritize external validation with larger and more diverse datasets to confirm the model’s robustness across settings. Also, the study used retrospective data and did not include external validation across multiple sites or years, limiting its ability to predict future or out-of-distribution cases. Future studies on this topic should also explore adaptive rule learning, richer natural language processing pipelines, and multi-jurisdictional validation to strengthen the model’s reliability and fairness.

## 5 Conclusion

This study demonstrates the successful implementation of a hybrid fuzzy logic–Random Forest model to predict the outcome of psychiatric treatment orders in Quebec’s Superior Court. By integrating structured demographic and legal variables with expert-informed semantic features and rule-based reasoning, the model achieved high predictive accuracy while preserving interpretability. The fuzzy inference system provided a transparent layer that mirrored clinical and legal judgment processes, while the machine learning classifier captured non-linear relationships and enhanced overall performance. The ability of the model to accurately distinguish between fully and partially accepted orders (while simultaneously offering an interpretable rationale for its predictions) addresses a need in both forensic psychiatry and legal medicine. Furthermore, the creation of the *Burden_Score* and *Expanded_Score* allowed for meaningful integration of clinical, behavioral, and procedural data, reinforcing the value of multidimensional modelling in such contexts. As health systems and courts increasingly turn to data-driven tools for support, this hybrid architecture offers a blueprint for responsible and context-sensitive AI deployment. Future research should aim to expand the dataset to include broader geographical coverage, refine semantic feature extraction with more sophisticated natural language processing techniques, and explore dynamic tuning of fuzzy rules through machine learning. With such advancements, hybrid models can serve not only as predictive tools but also as frameworks for dialogue between clinical judgment, legal reasoning, and artificial intelligence. This exploratory study paves the way for future research to enhance and deploy such models to verify their use in a real-world environment.

## Data Availability

The original contributions presented in the study are included in the article/Supplementary material, further inquiries can be directed to the corresponding author.
