# Supplementary material for: A hybrid fuzzy logic–Random Forest model to predict psychiatric treatment order outcomes: an interpretable tool for legal decision support
Source: Front Artif Intell. 2025 Jun 17;8:1606250. doi: 10.3389/frai.2025.1606250 (PMC12209287; doi:10.3389/frai.2025.1606250)
Supplement: Supplementary file 1 [file Data_Sheet_1.PDF]

A Hybrid Fuzzy Logic–Random Forest Model to Predict Psychiatric Treatment Order Outcomes: An Interpretable Tool for Legal Decision Support

Alexandre Hudon[1,2,3,4*]

[1]Centre de recherche de l'Institut universitaire en santé mentale de Montréal, Montreal, Quebec, Canada

[2]Institut universitaire en santé mentale de Montréal, Department of psychiatry, Montreal, Quebec, Canada

[3]Institut national de psychiatrie légale Philippe-Pinel, Department of psychiatry, Montreal, Quebec, Canada

[4]Université de Montréal, Faculty of Medicine, Department of psychiatry and addictology, Montreal, Quebec, Canada

Supplementary Material 2: Pseudocode

Pseudocode: Fuzzy + Hybrid Treatment Order Prediction Model

1. Load and Preprocess the Dataset

- Load dataset from Excel
- Drop identifier columns
- Convert categorical variables to numeric format
- Normalize continuous variables (e.g., age, time requested)
- Extract semantic flags from text fields:
-     - Severity_Flag = 1 if diagnosis suggests serious mental illness
-     - Compliance_Flag = 1 if symptoms suggest non-compliance

2. Create Composite Feature: Burden Score

- For each case:
-     Burden_Score = weighted average of:
-         35% * Severity_Flag
-         30% * Compliance_Flag
-         20% * Time Requested
-         15% * Substance Use

3. Apply Fuzzy Logic Rules to Generate Expanded Score

- For each case:
-     Initialize score = 0, weights = 0
-     Apply expert rules.
-     Expanded_Score = weighted average of all rule outputs

4. Split Dataset (70/30) for Training and Testing

- Use train_test_split() to split data into:
-     - 70% training set
-     - 30% testing set (hold-out for evaluation)

5. Apply k-Fold Cross-Validation

- Use StratifiedKFold from sklearn with k=10
- For each fold:
-     - Split training data into sub-train and validation
-     - Compute Burden_Score and Expanded_Score
-     - Train RandomForestClassifier on sub-train
-     - Validate on the fold and collect performance metrics
- Aggregate metrics across all folds

6. Train Final Random Forest Classifier

- Train on the entire training set using all features:
- \- Age, Sex, Legal Aid, Substance Use
- \- Time Requested, Time Granted
- \- Severity_Flag, Compliance_Flag
- \- Burden_Score, Expanded_Score

7. Evaluate on Test Set

- Predict labels and probabilities for test cases
- Compute evaluation metrics:
- \- Accuracy, Precision, Recall, F1 Score, ROC
- \- Confusion Matrix
- Generate visualizations (heatmaps, feature importances)

8. Interpret Predictions

- Use feature_importances_ to understand model decision logic
- Trace fuzzy rule activation per case for transparency
- Compare predicted labels to true outcomes for clinical validation

Summary

- This hybrid pipeline integrates explainable fuzzy logic with high-performing machine learning to predict mental health treatment order outcomes.
- It ensures interpretability, reproducibility, and empirical accuracy through structured rule logic and cross-validation.
